# Screening for Differentially Expressed Proteins Relevant to the Differential Diagnosis of Sarcoidosis and Tuberculosis

**DOI:** 10.1371/journal.pone.0132466

**Published:** 2015-09-14

**Authors:** Shan-Shan Du, Meng-Meng Zhao, Yuan Zhang, Peng Zhang, Yang Hu, Liu-Sheng Wang, Ying Zhou, Qiu-Hong Li, Yan Li, Yu-Kui Du, Xian He, Nan Li, Zhao-Fang Yin, Ya-Ru Wei, Dong Weng, Hui-Ping Li

**Affiliations:** 1 Department of Respiratory Medicine, Shanghai Pulmonary Hospital, Tongji University, School of Medicine, Shanghai, China; 2 Department of Chest Surgery, Shanghai Pulmonary Hospital, Tongji University, School of Medicine, Shanghai, China; 3 Department of Respiratory Medicine, Shanghai Pulmonary Hospital, Soochow University, School of Medicine, Suzhou, China; The Ohio State University, UNITED STATES

## Abstract

**Background:**

In this study, we sought to identify differentially expressed proteins in the serum of patients with sarcoidosis or tuberculosis and to evaluate these proteins as markers for the differential diagnosis of sarcoidosis and sputum-negative tuberculosis.

**Methods:**

Using protein microarrays, we identified 3 proteins exhibiting differential expression between patients with sarcoidosis and tuberculosis. Elevated expression of these proteins was verified using the enzyme-linked immunosorbent assay (ELISA) and was further confirmed by immunohistochemistry. Receiver operating characteristic (ROC) curve, logistic regression analysis, parallel, and serial tests were used to evaluate the diagnostic efficacy of the proteins.

**Results:**

Intercellular Adhesion Molecule 1(ICAM-1) and leptin were screened for differentially expressed proteins relevant to sarcoidosis and tuberculosis. Using ROC curves, we found that ICAM-1 (cutoff value: 57740 pg/mL) had an area under the curve (AUC), sensitivity, and specificity of 0.718, 62.3%, and 79.5% respectively, while leptin (cutoff value: 1193.186 pg/mL) had an AUC, sensitivity, and specificity of 0.763, 88.3%, and 65.8%, respectively. Logistic regression analysis revealed that the AUC, sensitivity, and specificity of combined leptin and ICAM-1 were 0.787, 89.6%, and 65.8%, respectively, while those of combined leptin, ICAM-1, and body mass index (BMI) were 0.837, 90.9%, and 64.4%, respectively, which had the greatest diagnostic value. Parallel and serial tests indicated that the BMI-leptin parallel with the ICAM-1 serial was the best diagnostic method, achieving a sensitivity and specificity of 86.5% and 73.1%, respectively. Thus, our results identified elevated expression of ICAM-1 and leptin in serum and granulomas of sarcoidosis patients.

**Conclusions:**

ICAM-1 and leptin were found to be potential markers for the diagnosis of sarcoidosis and differential diagnosis of sarcoidosis and sputum-negative tuberculosis.

## Introduction

Sarcoidosis is a granulomatous disease of unknown cause, characterized pathologically by noncaseous epithelioid granuloma. Sarcoidosis may cause damage to multiple organs, most commonly the lungs and intrathoracic lymph nodes [1]. The diversity of clinical manifestations and the lack of specificity make exclusive diagnosis the most effective way to identify sarcoidosis [2,3], which requires the exclusion of many other types of granulomatous diseases. The differential diagnosis of sputum-negative (smear- and culture-negative with sputum) tuberculosis is challenging but critical, especially for countries with high tuberculosis rates, such as China. Current strategies for the differential diagnosis of tuberculosis, however, have been limited to finding evidence for tuberculosis. Sarcoidosis-based biomarkers that can distinguish between these 2 diseases have not yet been identified. Additionally, while studies have identified several sarcoidosis-related serum markers, including soluble interleukin-2 receptor (sIL-2R), serum angiotensin-converting enzyme (SACE), and Krebs Von den Lungen-6 (KL-6) [4–6], these markers do not have sufficient sensitivity and specificity for the effective differential diagnosis of sarcoidosis, and the potential clinical applications of these markers have not been established.

In a previous study, we provided an alternative method for the diagnosis of tuberculosis and for distinguishing between sarcoidosis and tuberculosis: detection of *Mycobacterium tuberculosis* DNA in tissues by real-time polymerase chain reaction (PCR) [7]. In addition to infection-based molecular diagnosis, we established a multiparameter scoring system based on clinical-radiographical and histopathological data [8]. While great progress has been made in the differential diagnosis of sarcoidosis and sputum-negative tuberculosis, the accuracy of these methods is questionable, as evidenced by cases of false positives and false negatives. Moreover, these biopsy-based methods are subject to the uncertainties associated with different biopsy methods and the experience of the pathologist. Non–biopsy-based screening of serum amyloid A (SAA) differences using proteomics technology, as we previously established, could be used to diagnose sarcoidosis with a sensitivity of 96.3% but a specificity of only 52.5% [9]. Therefore, the discovery of novel serum markers for the diagnosis of sarcoidosis is critical, and it is essential to develop novel methods with better specificity, sensitivity, accuracy, and reliability for the differential diagnoses of sarcoidosis and sputum-negative tuberculosis.

The development of protein microarray technology [10] is considered a major milestone in proteomics, providing increasingly mature technologies to study changes in protein levels in various physiological and pathological circumstances. A study demonstrating the identification of diagnostic markers for the screening of patients with Alzheimer’s disease, published in *Nature Medicine* in 2007, supported the validity of protein microarray technology for biomarker screening [11]. Currently, there are several protein microarrays commercially available for clinical use, such as protein chip systems for parallel analysis of multiple tumor markers [12].

In this study, we sought to identify proteins that were differentially expressed between patients with sarcoidosis and tuberculosis using protein microarray and to improve the sensitivity and specificity of the differential diagnosis of these 2 diseases. Our study provides new directions for research on the pathogenesis of sarcoidosis.

## Methods and Materials

Ethical approval for this study was obtained from the Human Research Ethics Committee, Tongji University School of Medicine and Life Science (2011-FK-10). Written informed consent was obtained from all patients involved in this study.

The individuals in this manuscript provided written informed consent (as outlined in the PLOS consent form) to allow for publishing of the case details contained in this manuscript.

### Patients and diagnostic criteria

#### Patients and controls

For protein microarray analysis, a total of 60 participants were divided into 3 groups. The sarcoidosis (SA) group included 20 participants who had been diagnosed using a biopsy, among which, 4 were diagnosed from lung tissues, 12 from lymph nodes, and 4 from subcutaneous nodules. The tuberculosis (TB) group comprised 20 hospitalized patients with newly diagnosed smear-positive pulmonary tuberculosis at Shanghai Pulmonary Hospital from September 2011 to April 2012. The healthy control (HC) group included 20 healthy individuals who had physical examinations at the same hospital from January to April 2012 (shown in Table 1).

**Table 1:** General information and characteristics of the SA, TB, and HC groups included in the protein microarray analysis.

	SA*^a^	TB*^a^	HC*^a^
	(n = 20)	(n = 20)	(n = 20)
Age, years	49.8 ± 9.9	40.7 ± 16.2	30.1 ± 8.6
Male/female ratio	4/16	15/5	17/3
Biopsy	4/12/4	N/A*^b^	N/A*^b^
(lung/lymphonodus/skin)			

*a SA = sarcoidosis, TB = tuberculosis, HC = healthy control.

*b N/A = not applicable.

**Fig 1:**
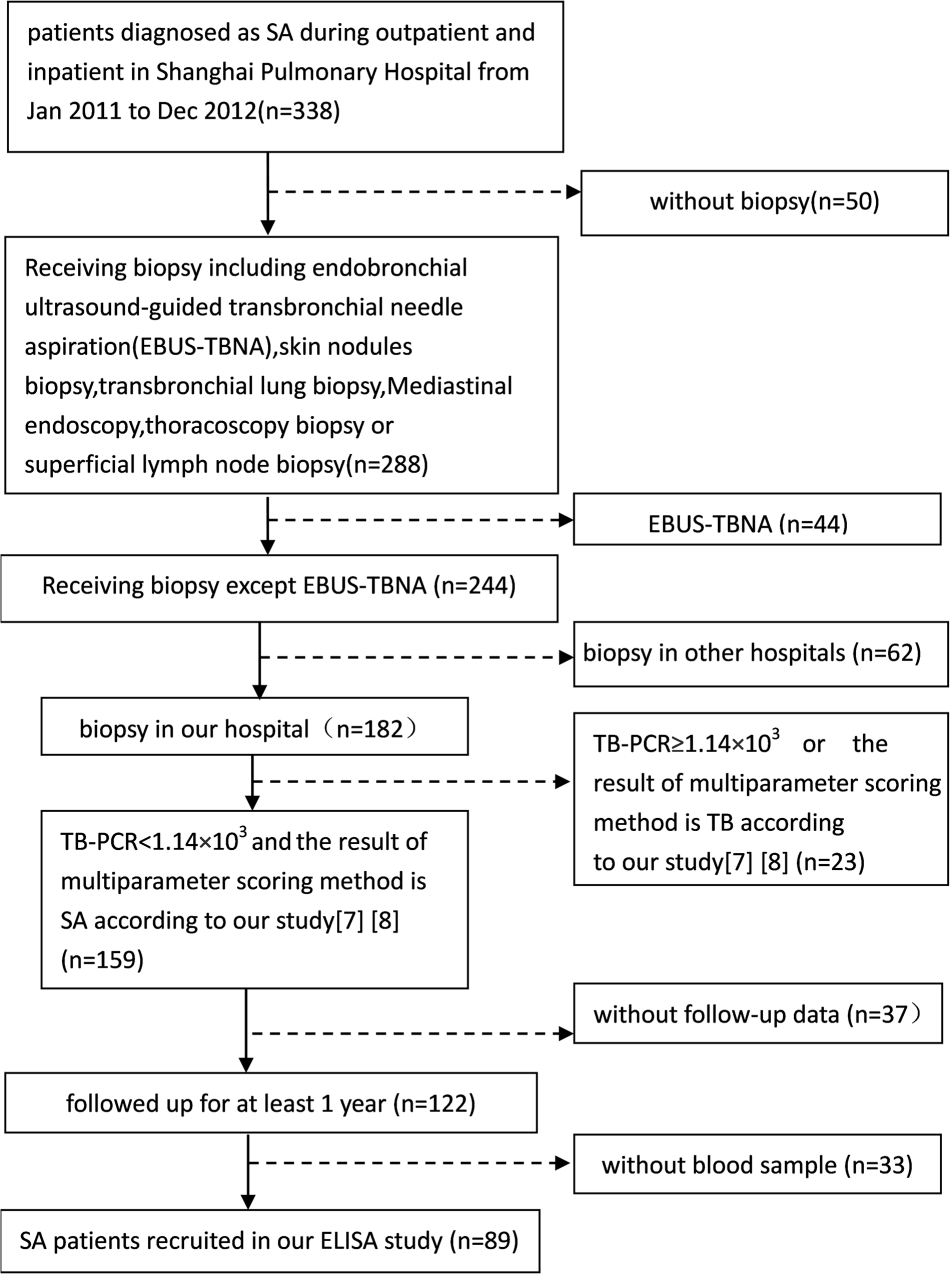
Procedure for recruiting SA patients.

**Fig 2:**
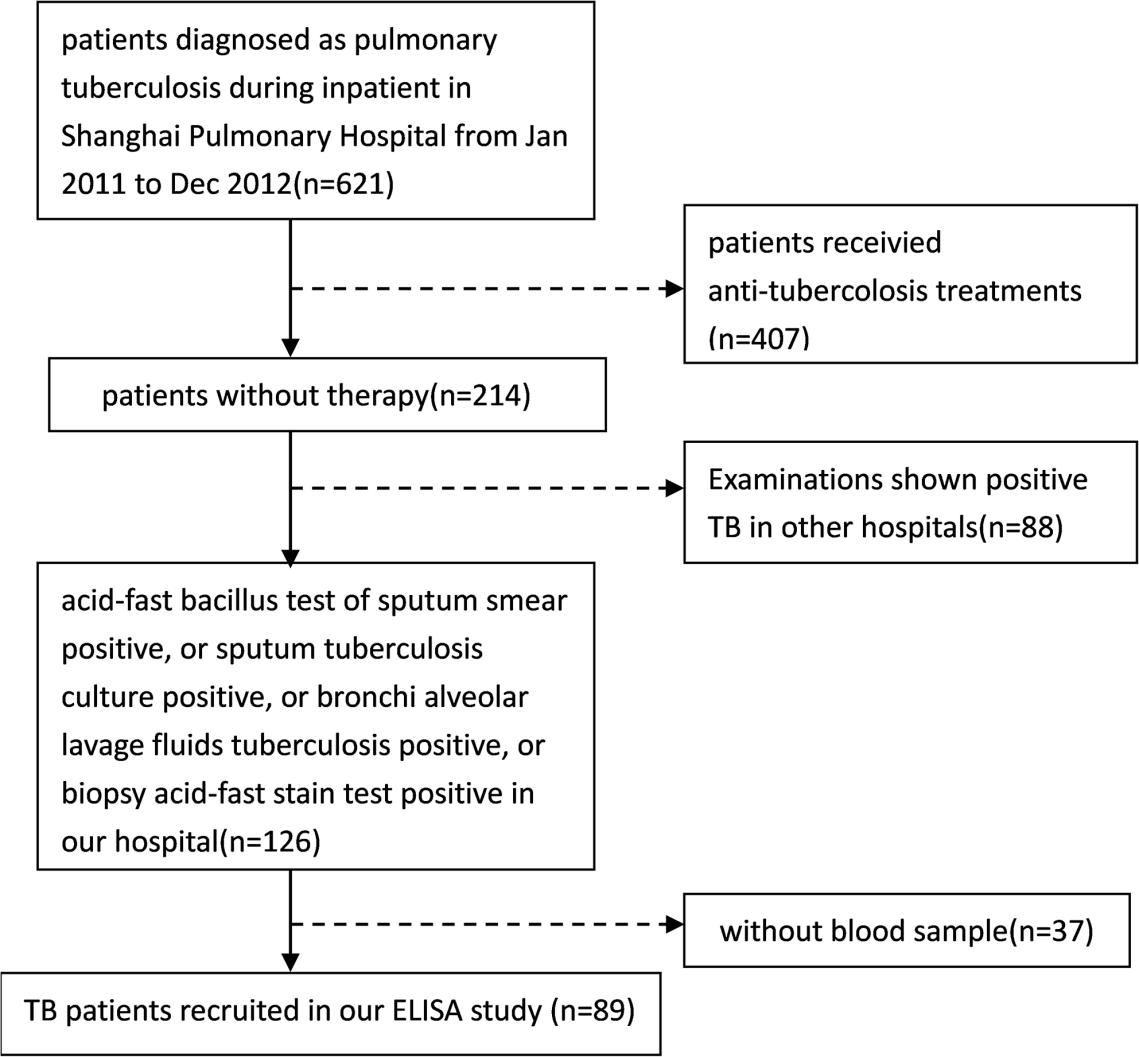
Procedure for recruiting TB patients.

The procedures for recruiting participants in the sarcoidosis and tuberculosis groups for the enzyme-linked immunosorbent assay (ELISA) are shown Figs 1 and 2, and the HC group for ELISA analysis was selected from the physical examination center in our hospital from January 2011 to December 2012.

#### Diagnostic criteria

Diagnosis of sarcoidosis followed the ATS/ERS/WASOG criteria (3). Any patients with a biopsy that showed noncaseous necrotizing granulomas and other granulomatous diseases (tuberculosis, parasitic infections, fungal infections, tumors, vasculitis, etc.) were excluded. In addition, sputum smear-negative fertile tuberculosis was excluded using TB-PCR [7] and the multiparameter scoring method [8]. Follow up for all patients continued for at least 1 year.

Diagnosis of tuberculosis was determined using the guidelines for tuberculosis diagnosis and treatment amended by the Chinese Society for Tuberculosis of the Chinese Medical Association in 2001. The following positive test results were required: acid-fast bacillus test of sputum smear, sputum tuberculosis culture, bronchi alveolar lavage fluids tuberculosis, or biopsy acid-fast stain test. All patients were treated with anti-tuberculosis therapy.

### Serum acquisition

Serum collection was carried out as described in the Human Proteome Organization Plasma Proteomics Project [13]. Serum samples were stored at -80°C before use.

### Differential expression analysis using protein microarray

We collected 3 serum samples from patients with sarcoidosis and 2 serum samples from healthy donors and compared the expression of 120 cytokines and chemokines using a RayBiotech Human Cytokine Antibody Array G Series 1000 (see S1 Table). Based on the results and published papers, we customized the protein microarray (RayBiotech QAH-CUST, Guangzhou, China) with 30 key proteins (see S1 File). Twenty serum samples from each group were analyzed using a Genepix fluorescence scanner (Axon, United States) according to the manufacturer’s instructions. Fluorescence signals were collected via the Cy3 channel, and data were analyzed using QAH-CUST-G software.

### Verification of differentially expressed proteins using ELISA

The primary screening result from the protein microarray was further verified using additional samples. Intercellular Adhesion Molecule 1(ICAM-1), leptin, and platelet-derived growth factor (PDGF-BB) levels were measured using ELISA kits (Neobioscience Technology, China), and the optical density at 450 nm was detected using an ELX808 microplate reader (BioTek Instruments, USA).

### Detection of differentially expressed proteins using immunohistochemistry (IHC)

Differentially expressed proteins were detected in biopsy specimens of granulomatous tissue and lymph nodes using Strept Avidin-Biotin Complex IHC. Biopsy specimens, including those from 4 lung tissues and 14 lymph nodes in the SA group and 3 lung tissues and 9 lymph nodes in the TB group, were embedded in paraffin and sectioned to 5-μzm thickness. Adhesion microscope slides were used to prevent sections from escaping. The experiments were carried out according to the instructions for the IHC kit (Vector Laboratories, USA). Diaminobenzidine reagent was purchased from Neobioscience Technology, anti-leptin antibodies were from Abcam (UK), and anti-ICAM-1 antibodies were from Epitomics (USA).

### Statistical analysis and establishment of the differential diagnosis model

All experiments were performed in triplicate. Data were statistically analyzed using Graphpad Prism5 and SPSS v. 19.0 and are presented as the means ± standard deviations (SDs). Independent-samples t tests, assuming equal variances, and one-way analysis of variance (ANOVA) were used for microarray and ELISA data comparisons between the SA and HC groups. Post hoc tests of one-way ANOVA were performed with the least-significant difference (LSD) and Student-Newman-Keuls (S-N-K) tests for data with equal variances or the Tamhane test for data with unequal variances. Correlation analysis and regression analysis were used to analyze plasma concentrations in association with BMI and leptin levels, and analysis of covariance was performed to eliminate the impact of BMI on leptin concentrations in serum samples. Receiver operating characteristic (ROC) curves of ELISA data were plotted using SPSS v. 19.0 software, and quantification was based on the optimal cutoff value with the maximum Youden index (Youden index = sensitivity + specificity—1). Combination diagnosis was evaluated with a prediction equation constructed by logistic regression, and serial and parallel tests were performed to verify the accuracy of diagnosis. Differences or associations with *P* values of less than 0.05 were considered statistically significant.

## Results

### General information and characteristics of the sarcoidosis and control groups (Tables 1 and 2)

**Table 2:** General information and clinical indices of the SA, TB, and HC groups included in the ELISA analysis.

	SA^*d^	TB^*d^	HC^*d^
	(n = 89)	(n = 89)	(n = 91)
Age, years	47.55 ± 11.89	39.75 ± 16.63	30.0 ± 7.3
Male/female ratio	26/63	55/34	50/41
CXR stage*^a^	0/33/39/10/7	N/A	N/A
0/I/II/III/IV			
Tuberculosis examination	89/0	0/89	0/0
negative/positive			
BMI (kg/m^2^)*^b^	23.37 ± 2.97 (89)	19.93 ± 2.93(89)	24.10 ± 2.56(91)
SACE (IU/L)	79.8 ± 50.2 (89)	/	/
ESR (mm/h)*^c^	18.9 ± 12.8 (63)	48.9 ± 33.01 (84)	/
24 h urinary calcium	6.59 ± 2.84 (65)	/	/
IL-1 (ng/L)	32.0 ± 25.0(38)	43.86 ± 59.13 (14)	/
IL-2R (pmol/mL)*^c^	177.1 ± 92.5 (38)	114.0 ± 49.25 (14)	/
IL-5 (ng/L)*^c^	33.6 ± 30.1 (38)	57.0 ± 35.13 (14)	/
IL-6 (ng/L)	68.9 ± 154.5 (38)	43.43 ± 42.03 (14)	/
IFN-r (ng/L)	23.7 ± 14.0 (38)	31.21 ± 55.28 (14)	/
TNF (ng/L)	48.8 ± 32.1 (38)	49.29 ± 16.37 (14)	/
CD3 (%)	32.2 ± 32.7 (65)	36.84 ± 34.31 (68)	/
CD4 (%)	18.9 ± 19.4 (65)	21.12 ± 20.36 (68)	/
CD8 (%)	11.1 ± 12.7 (65)	11.4 ± 11.52(68)	/
IgG (ng/L)	12.8 ± 3.1 (58)	13.57 ± 4.521 (54)	/
IgA (ng/L)	2.3 ± 0.9(58)	2.819 ± 1.898 (54)	/
IgM (ng/L)	1.22 ± 0.64 (58)	1.14 ± 0.7337 (54)	/
C3 (ng/L)	1.15 ± 0.22 (58)	1.169 ± 0.2857 (54)	/
C4 (ng/L)	0.32 ± 0.09 (58)	0.3583 ± 0.1232 (54)	/

*a. CXR = chest X-ray; N/A = not applicable; CXR stage: 0 = no adenopathy, no lung infiltrates; Stage I = hilar and mediastinal adenopathy only; Stage II = hilar and mediastinal adenopathy plus lung infiltrates; Stage III = lung infiltrates only; Stage IV = pulmonary fibrosis.

*b. BMI (body mass index) = weight/height^2^, SA vs. TB (*P* < 0.00001), SA vs. HC (*P* > 0.05).

*c. SA vs. TB (*P* < 0.05).

*d SA = sarcoidosis, TB = tuberculosis, HC = healthy control.

For the protein chip study, we collected serum from 60 patients: 20 with sarcoidosis, 20 with tuberculosis, and 20 healthy controls. Additionally, ELISA was performed using the serum from 89 patients with sarcoidosis, 89 patients with positive tuberculosis, and 91 healthy controls. IL-2R, IL-5, and erythrocyte sedimentation rate (ESR) differed significantly between the SA and TB groups. IL-2R and IL-5 differed dramatically between patients in the SA and TB groups.

### Screening for the differential expression of ICAM-1, leptin, and PDGF-BB proteins by protein microarray (Tables 3 and 4)

**Table 3:** Serum protein levels in the SA, TB, and HC groups, as measured by protein microarray analysis (mean ± SD).

(pg/mL)	SA*	TB*	HC*
BMP-7	44874.75±21445.49	57917.5±30044.48	35503.55±28650.11
EOT	397.2±224.2392	337.95±248.6425	497.55±780.2128
F1t-3L	456.2±105.0837	446.45±92.97338	360.2±96.55737
G-CSF	211.5±198.1856	301.2±178.3121	146.5 ±200.4659
GM-CSF	1262.15±556.0675	1327.85±516.2684	875.4±313.4884
ICAM-1	253221.3±176473.4	130344.3±127061	198636.1±155172.6
IFNg	5611.05±2298.708	6072±2072.293	3791.65±1554.821
IL-10	1497.4±551.5003	1645±577.0071	1100±406.4593
IL-12 p40	5258±1843.054	6125.7±2469.264	4332.3±3231.537
IL-12 p70	1081.35±460.93	1330.85±588.6443	917.95±981.7312
IL-13	795.15±402.1676	861±361.0183	549.95±263.7474
IL-15	2982±1083.346	3201.95±1238.03	1947.9±764.2845
IL-17	1904.35±944.0118	2416.65±1349.232	1016.25±669.5217
IL-1a	186.35±104.5327	221.35±97.49131	183.95±304.0295
IL-1b	131.7±88.18288	215.9±174.4024	179.1±389.4466
IL-22	3581.7±15068.23	9428±25657.45	82083.75±320987.2
IL-4	34.45±42.17691	41.75±24.88896	23.75±39.65227
IL-6	940.05±398.233	988.3±347.8752	630.05±319.0381
IL-7	389.7±151.8535	417.25±123.2874	311.8±111.0332
IL-9	125966±42052.17	168476.9±84660.55	74035.75±50073.08
Leptin	421491.2±314492	140270.5±224479.3	122349.5±160519.4
LIGHT	6249.4±8592.651	15719.2±25053.21	55054.95±193183.7
MCP-1	317.9±166.5428	296.7±145.3247	181.7±99.13367
MCP-2	59.9±39.50203	39.65±55.59655	107.35±255.4359
MIG	4057.05±1854.478	3712.35±1489.822	1385.65±698.4668
NT-3	263.1±152.2463	379.25±272.468	318.45±825.8245
MMP-7	/	/	/
PDGF-BB	3465.95±1232.81	2704.8±950.945	3463.8±1479.256
TGF-b1	129496.7±54709.25	140587±63692.1	77560.4±38565.38
TSLP	319.75±244.2893	420.2±373.4545	311.05±605.729

“/” indicates not detected.

* SA = sarcoidosis, TB = tuberculosis, HC = healthy control.

**Table 4:** Identification of differentially expressed proteins in the SA and TB groups using protein microarray analysis.

	HC^*a^ vs. SA^*a^	HC vs. TB^*a^	SA vs. TB
*(pg/mL)*	*P* value	*P* value	*P* value
BMP-7	0.248865	0.020679	0.122358
EOT	0.583624	0.388886	0.433631
F1t-3L	0.004642	0.006541	0.757677
G-CSF	0.308966	0.013916	0.140663
GM-CSF	0.010052	0.001835	0.700749
ICAM-1	0.305458	0.136086	**0.015785** *^b^
IFNg	0.005677	0.000341	0.509389
IL-10	0.013397	0.001375	0.413405
IL-12 p40	0.272779	0.055912	0.215573
IL-12 p70	0.504531	0.114987	0.143841
IL-13	0.028303	0.003527	0.588999
IL-15	0.001246	0.000434	0.553437
IL-17	0.001461	0.000176	0.17222
IL-1a	0.973543	0.603416	0.28039
IL-1b	0.598598	0.701885	0.061506
IL-22	0.281482	0.319331	0.385091
IL-4	0.41362	0.093669	0.509039
IL-6	0.009866	0.001622	0.68551
IL-7	0.071817	0.007166	0.532533
IL-9	0.001041	0.000117	0.05144
Leptin	0.000525	0.773077	**0.002387** *^b^
LIGHT	0.266093	0.37219	0.118108
MCP-1	0.00324	0.005804	0.670392
MCP-2	0.416774	0.254023	0.192149
MIG	5.2E-07	2.05E-07	0.520858
MMP-7	/	/	/
NT-3	0.769772	0.756237	0.104291
PDGF-BB	0.996042	0.061071	**0.035023** *^b^
TGF-b1	0.001312	0.000531	0.558216
TSLP	0.95281	0.496899	0.320469

*a SA = sarcoidosis, TB = tuberculosis, HC = healthy control.

*b *P* < 0.05; “/” indicates not detected.

For the protein chip study, we collected serum from 60 patients (20 with sarcoidosis, 20 with tuberculosis, and 20 healthy controls) and analyzed the expression of 30 cytokines and chemokines. Three of them were differentially expressed in the SA and TB groups.

### Analysis of differences in protein expression using ELISA

**Fig 3:**
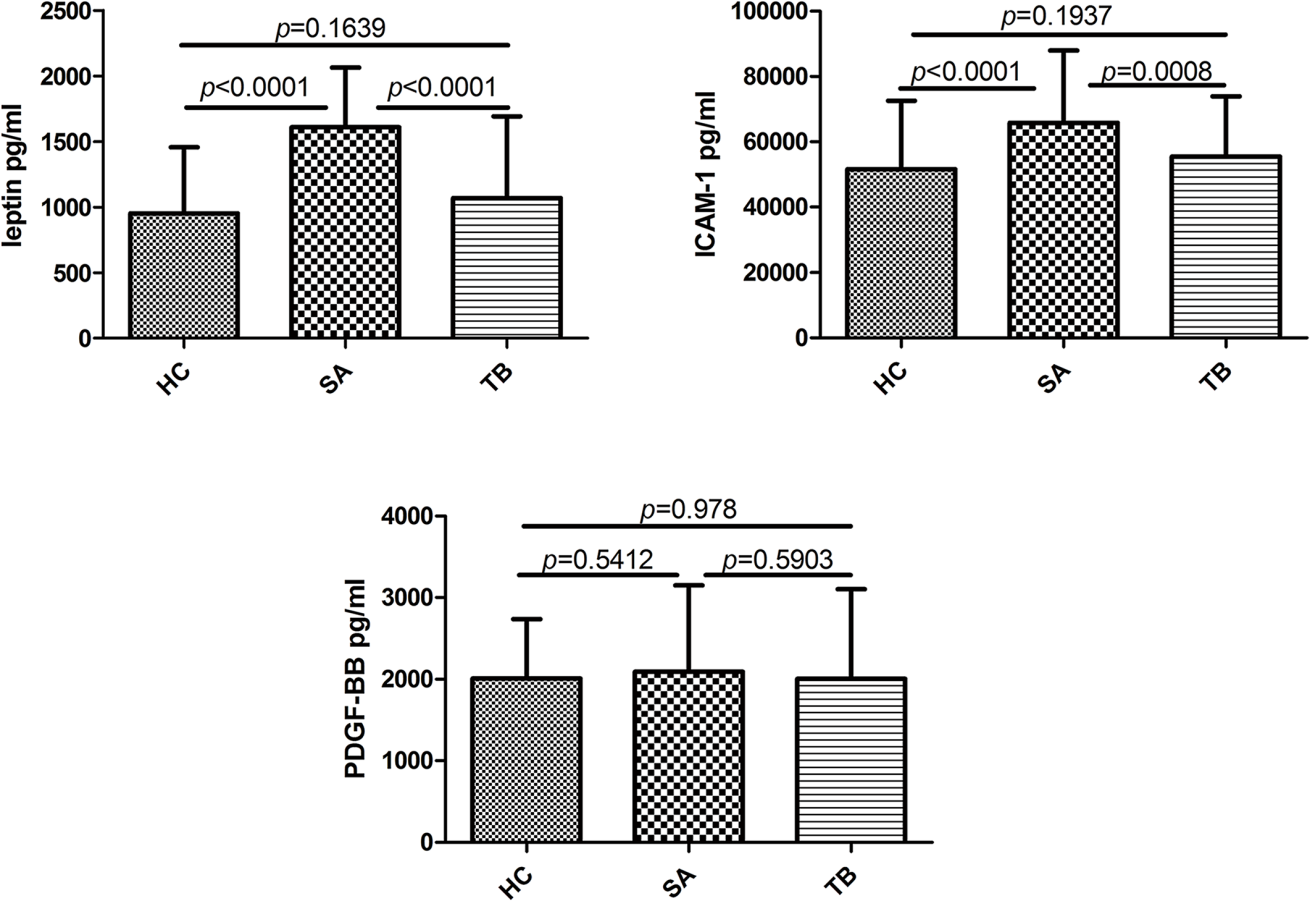
Differences between groups analyzed by one-way ANOVA and post hoc tests. Levels of leptin, ICAM-1, and PDGF-BB were assayed in healthy patients and patients with sarcoidosis or tuberculosis. Statistical significance is indicated. Error bars denote S.D. The results showed that the expression levels of leptin and ICAM-1 in the SA group were significantly different from those in the HC and TB groups, respectively (*P* < 0.001). Interestingly, no significant differences were observed between the HC and TB groups (*P* > 0.05). Additionally, there were no significant differences in PDGF-BB expression levels among the 3 groups (*P* > 0.05).

Differences in the expression levels of leptin, ICAM-1, and PDGF-BB in each group were evaluated using ELISA with additional samples (Fig 3). The expression levels of leptin and ICAM-1 in the SA group were significantly different from those in the HC and TB groups (*P* < 0.001). Interestingly, no significant differences in expression levels of leptin and ICAM-1 were observed between the HC and TB groups (*P >* 0.05). Additionally, there were no significant differences in PDGF-BB expression levels among the 3 groups (*P >* 0.05).

The correlation analysis showed that the concentration of leptin in the serum was significantly correlated with body mass index (BMI; *P* < 0.0001), with a Pearson correlation coefficient of r = 0.549. Regression analysis showed that there was a positive, linear relationship between BMI and leptin concentration (*P* < 0.001), which was consistent with a previous report demonstrating that leptin expression was related to body weight. In order to exclude the influence of body weight, covariance analysis was performed to analyze changes in leptin expression in the SA and TB groups using BMI as the covariant component. After correction, the *P* value for the difference in leptin concentrations between the SA and TB groups was 0.013, suggesting that there was still significant difference between the groups after adjusting for BMI.

### Test of granuloma specimens using IHC

**Fig 4:**
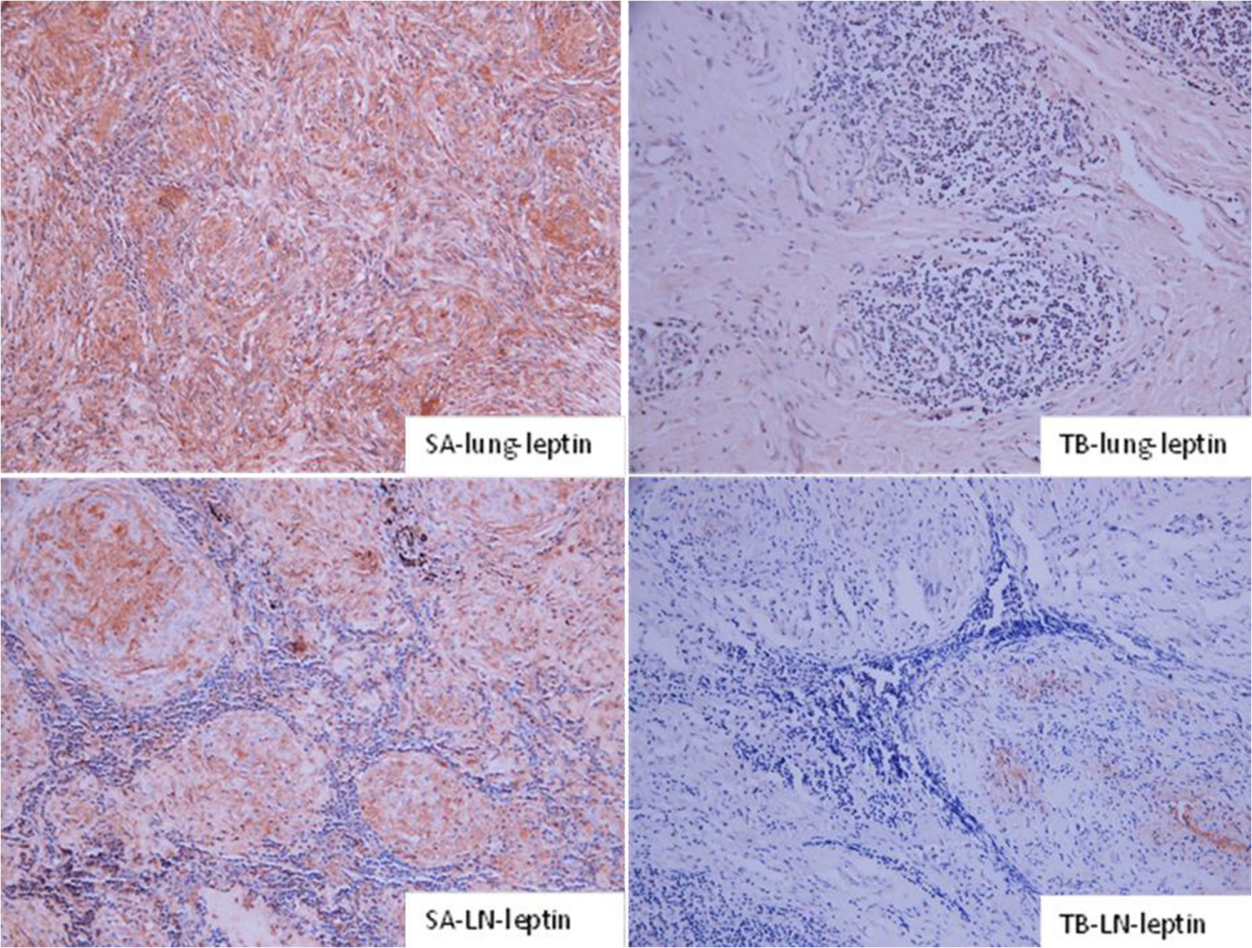
IHC analysis of lung sections from patients in the SA and TB groups. Staining for leptin is shown (×200). In the SA group, leptin exhibited positive expression in the lung and was scattered in the granuloma and its adjacent tissues. In the TB group, it showed nonspecific staining, although some hazel-colored regions could be imaged in the adjacent tissues. Leptin accumulated in the center of the lymphoglandulae granuloma in the SA group, exhibiting strong positive expression, while that in the TB group showed significantly weaker expression (Fig 2).

**Fig 5:**
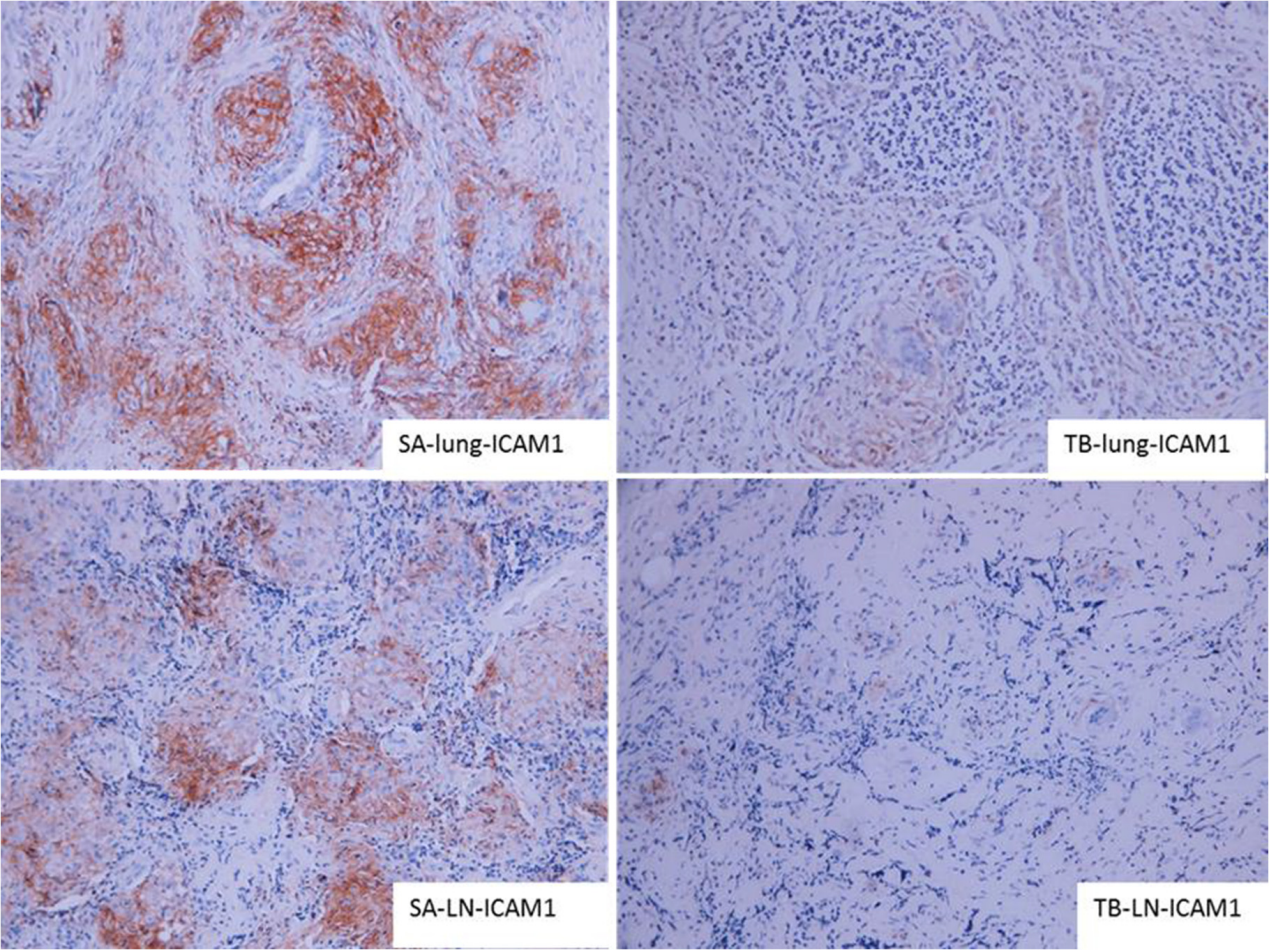
IHC analysis of lung sections from patients in the SA and TB groups. Staining for ICAM-1 is shown (×200). ICAM-1 showed scattered, clustered, strong expression in the lungs and lymph nodes of patients in the SA group but exhibited nonspecific staining or weak expression in the lungs of patients in the TB group. Additionally, a hazel-colored region was observed in the tissues adjacent to the granuloma (Fig 3).

The positive expression of leptin and ICAM-1 in the lungs and mediastinal lymph nodes in the SA group was significantly higher than that in the TB group (Figs 4 and 5), indicating that these 2 proteins could be used as markers for TB diagnosis.

### Leptin and ICAM-1 in the differential diagnosis of SA and TB: preliminary evaluation of ROC curves and efficacy of differential diagnosis models

**Fig 6:**
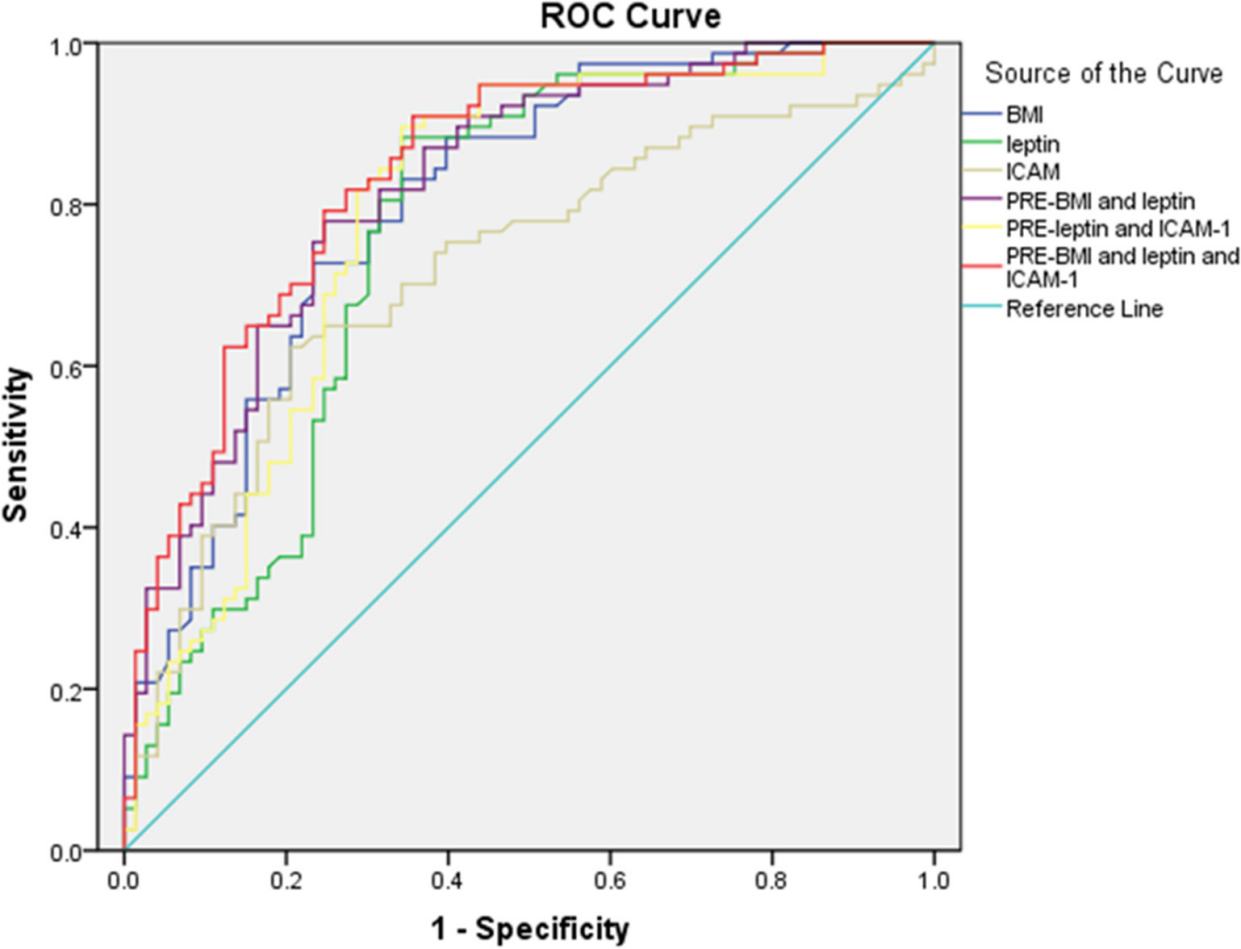
ROC curves for the association of BMI, leptin, and ICAM-1 or combined factors with the differential diagnosis of SA and TB. The concentrations of serum ICAM-1 and leptin obtained from ELISA analysis were used to establish ROC curves to identify SA (n = 89) and TB (n = 89).

The concentrations of serum ICAM-1 and leptin obtained from the ELISA analysis were used to establish ROC curves to identify sarcoidosis (n = 89) and tuberculosis (n = 89; Fig 6). Cutoff values were established when the Youden index reached the maximum (Tables 5 and 6).

**Table 5:** AUCs of the ROCs and 95% confidence intervals for leptin and ICAM-1 in the differential diagnosis of SA and TB.

				Asymptotic	
				95% confidence interval	
Test result variable(s)	Area	Std. error*^a^	Asymptotic sig.*^b^	Lower bound	Upper bound
Leptin	0.763	0.04	0	0.684	0.842
ICAM	0.718	0.042	0	0.635	0.801
PRE*^c^-BMI and leptin	0.821	0.034	0	0.755	0.887
PRE-leptin and ICAM-1	0.787	0.039	0	0.712	0.863
PRE-BMI, leptin, and ICAM-1	0.837	0.033	0	0.773	0.901

*a. Under the nonparametric assumption.

*b. Null hypothesis: true area = 0.5.

*c.PRE = prediction probability.

**Table 6:** Cut-off values, sensitivity, and specificity of ROC curves for differential diagnosis of SA and TB.

Detection index	Diagnostic cut-off point	Sensitivity (%)	Specificity (%)
	(sarcoidosis if greater than or equal to)		
Leptin (pg/mL)	1193.186	88.3	65.8
ICAM-1 (pg/mL)	57740	62.3	79.5
PRE-BMI and leptin*^a^	0.5076	0.779	0.753
(probability)			
PRE-leptin and ICAM-1*^b^	0.408	89.6	65.8
(probability)			
PRE-BMI, leptin, and ICAM-1*^c^ (probability)	0.3651	90.9	64.4

*a. PRE-BMI and leptin: When we applied BMI and leptin to the logistic regression equation, if the prediction probability was positive (greater than or equal to the cutoff value: 0.5076), patients were diagnosed with sarcoidosis.

*b. PRE-leptin and ICAM-1: When we applied leptin and ICAM-1 to the logistic regression equation, if the prediction probability was positive (greater than or equal to the cutoff value: 0.4080), patients were diagnosed with sarcoidosis.

*c. PRE-BMI, leptin, and ICAM-1: When we applied BMI, leptin, and ICAM-1 to the logistic regression equation, if the prediction probability was positive (greater than or equal to the cutoff value: 0.3651), patients were diagnosed with sarcoidosis.

The area under the curve (AUC), sensitivity, and specificity of ICAM-1 (cutoff value: 57740 pg/mL) were 0.672, 87.6%, and 62.6%, respectively, while those of leptin (cutoff value: 1193.186 pg/mL) were 0.742, 60.7%, and 70.3%, respectively. Next, we combined these prediction factors to construct equations with logistic regression. The AUC, sensitivity, and specificity of the combined prediction factors (ICAM-1 and leptin) from the derived equation were 0.760, 88.8%, and 62.6%, respectively. Since patients with sarcoidosis and tuberculosis had significantly different BMIs, BMI was added into the regression equation as a third prediction factor, yielding an AUC, sensitivity, and specificity of 0.837, 90.9%, and 64.4%, respectively. The AUC was higher after adding in BMI than that obtained for any other condition, indicating that BMI had the highest diagnostic significance. The null hypothesis was excluded (AUC = 0.5).

The AUC of leptin (0.763) was higher than that of ICAM-1 (0.718), suggesting that the diagnostic value of leptin for these 2 diseases was higher than that of ICAM-1. When BMI together with leptin or BMI and leptin combined with ICAM-1 were used for constructing logistic regression equations for differential diagnosis, the AUCs (0.821 and 0.837) were all higher than when each factor was used alone (0.801, 0.763, and 0.718, respectively), indicating that the combined application of the indices was more reliable in establishing logistic regression equations for SA and TB differential diagnosis.

In clinical practice, parallel and serial tests are usually performed by the combination of multiple indices. In the parallel test, also known as a comparative experiment, the results are identified as positive when 1 of the 2 indices is positive, which improves sensitivity and reduces the rate of misdiagnosis. In contrast, in the serial test, also known as the sequential test, the results are identified as positive only when 2 or more indices are positive, which improves specificity and reduces the rate of misdiagnosis. In this study, the difference in the BMIs between patients in the SA and TB groups were positively correlated with the concentration of leptin. Thus, BMI and leptin were combined for a serial test. When the BMI was greater than 21.6 kg/m^2^ and leptin was greater than 1193.186 pg/mL (Table 7), leptin was used as the sole index for differential diagnosis, with a specificity of 92.0% and a sensitivity of 64.2%. When the BMI was greater than 21.6 kg/m^2^, leptin was greater than 1193.186 pg/mL, and ICAM-1 was greater than 57740 pg/mL, BMI and leptin were used for serial tests, with a specificity of 96.9% and a sensitivity of 40%. However, when the serial combination of BMI and leptin as one factor was used in combination with ICAM-1 for the differential diagnosis, the specificity increased to 73.1% and the sensitivity reached 86.5%, providing ideal results.

**Table 7:** Sensitivity and specificity of the parallel and serial tests for differential diagnosis of SA and TB.

Detection index	Diagnosis cut-off point (sarcoidosis if greater than or equal to)	Sensitivity (%)	Specificity (%)
Parallel test*^a^(leptin or ICAM-1)	Leptin > 1193.186 or ICAM-1 > 57740	96	52.3
Serial test*^b^(BMI and leptin)	BMI > 21.6 and leptin > 1193.186	64.2	92
Serial test(leptin and ICAM-1)	Leptin > 1193.186 and ICAM-1 > 57740	55	86.6
Parallel test(BMI and leptin or ICAM-1)	BMI > 21.6 and leptin > 1193.186 or ICAM-1 > 57740	86.5	73.1
Serial test(BMI, leptin, and ICAM-1)	BMI > 21.6 and leptin > 1193.186 and ICAM-1 > 57740	40	96.9

*a. Parallel test: If one of the indices was positive (greater than or equal to the cutoff value), the patient was diagnosed with sarcoidosis.

*b. Serial test: If both of the indices were positive (greater than or equal to the cutoff value), the patient was diagnosed with sarcoidosis.

## Discussion

The differential diagnosis of sarcoidosis and sputum-negative tuberculosis has always been difficult due to their similarities in clinical manifestations, imaging, and pathology. This issue is critical for countries that have high tuberculosis rates, such as China in which 40%–60% of pulmonary tuberculosis cases are sputum-negative. A national epidemiological sampling survey in 2000 showed that 2 million people suffer from sputum-negative tuberculosis in China each year [14]. Because misdiagnosis can lead to the patient receiving the incorrect treatment and could lead to an outbreak of tuberculosis, it is essential to develop methods for differential diagnosis with excellent sensitivity and specificity. Real-time PCR and multiparameter scoring [7,8], 2 alternative methods we developed previously, have sensitivities and specificities of over 90%; however, they both depend on biopsy samples, thereby limiting their applications. SAA has been identified by the proteomics clinprot system as showing potential as a serum marker for sarcoidosis [9] and differential diagnosis, but the specificity of this marker is not optimal. Arakelyan et al. [15] detected 24 inflammatory molecules from bronchoalveolar lavage fluid from acute sarcoidosis (Löfgren’s syndrome) and Stage III sarcoidosis patients using protein microarray technology and found that chemokine C-C motif ligand 15 (CCL15) and macrophage-stimulating protein (MSP) were likely correlated with the pathogenesis of sarcoidosis. However, due to the invasiveness of bronchoalveolar lavage and the lack of stability and repeatability of the assay, this method is also not applicable for use in clinical diagnosis.

Based on our previous research, we took advantage of the efficiency of protein microarray technology [10] and screened a large number of proteins. We identified 2 differentially expressed proteins, leptin and ICAM-1, for the differential diagnosis of sarcoidosis and sputum-negative tuberculosis. Subsequent ELISA analysis using additional samples and IHC analysis of pathological specimens verified the significant differences in leptin and ICAM-1 expression.

Leptin, encoded by the so-called obesity gene, is a 16-kD nonglycosylated protein hormone secreted by adipocytes [16,17]. Mainly functioning to suppress the appetite and regulate energy metabolism, leptin has been shown to play important roles in various processes, such as reproduction, the immune response, hematopoiesis, and angiogenesis. Leptin is responsible for the activation of the T helper type 1 (Th1) response via upregulation of IL-2 and interferon (IFN)-r [18]. Overactivation of Th1 may lead to the formation of granulomas, consistent with the hypothesized formation of granulomas in SA [19]. Leptin directly activates monocytes and further activates costimulated T cells, inducing a Th1 response that amplifies the pro-inflammatory response [20].

Serum leptin concentrations are positively related to BMI [21]. In our study, the BMI of the SA group was significantly different from that of the TB group (Table 2); therefore, we had to rule out the potential effects of BMI on leptin expression. Our analysis with BMI as a concomitant variable showed that leptin expression in the SA group was significantly elevated compared to that of the TB group, while no significant difference was observed between the SA and HC groups. Therefore, we confirmed the upregulation of serum leptin in sarcoidosis patients, which suggests that leptin may be a novel diagnostic marker. Previous studies on the relationship between plasma leptin and sarcoidosis or tuberculosis have been controversial [22–24]. A study by Yurt et al. detected peripheral plasma leptin in sarcoidosis patients and healthy individuals using ELISA and found that leptin was downregulated in tuberculosis patients, without substantial changes observed in sarcoidosis patients [22]. Cakir et al. found increased leptin levels in patients with active pulmonary tuberculosis [23], while Crevel et al. observed the opposite trend [24]. These contradictions may be explained in part due to the differences in the number of samples, method of detection, and preparation of samples. In our study, we used serum samples to avoid cross-interaction of plasma proteins and adopted accurate and sensitive protein microarray and ELISA methods for quantification. Together with our adequate number of samples, we are confident of the reliability of our results and their potential value in clinical practice.

ICAM-1, also known as CD54, is a member of the immunoglobulin superfamily (IGSF), first discovered as a ligand of leukocyte function-associated antigen-1 (LFA-1) [25]. ICAM-1 is required for cell adhesion and plays an important role in inflammation-induced tissue adhesion, tumor metastasis, and regulation of immune response. The binding of ICAM-1 to specific receptors increases the adhesion of inflammatory cells and tumor cells to endothelial cells, activates endothelial cells, and facilitates penetration into inflammatory tissue. There are 2 forms of ICAM-1: the soluble form (sICAM-1) and the membrane form (mICAM-1). Serum sICAM-1 could be used as an indicator of ICAM-1 levels in local tissue. As early as 1993, ICAM-1 was reported to be distributed widely in the vascular endothelium and pulmonary granulation tissue in sarcoidosis patients [26], consistent with our IHC experimental results. In 1995, Ishii et al. [27] observed high concentrations of ICAM-1 in the alveolar lavage fluid of patients with active-stage sarcoidosis. Additionally, in 1999, Kim et al. [28] detected concentrations of ICAM-1 in the serum and alveolar lavage fluid of sarcoidosis patients during disease development and follow-up and found that ICAM-1 levels increased as sarcoidosis progressed. In our study, we found that ICAM-1 levels were relatively higher in the TB group than those in the HC group, consistent with the previous report. Moreover, Hamzaoui et al. [29] found that serum ICAM-1 concentrations were higher in patients with tuberculosis (n = 15) than in healthy controls (n = 20), which contrasted with our study, in which we observed nonsignificant differences in ICAM-1 expression between the TB and HC groups. This difference may have been because our study had fewer cases than the study by Hamzaoui et al. In terms of the mechanism and relevance of the high expression of ICAM-1 in sarcoidosis patients, current research has demonstrated that alveolar macrophages associated with the sarcoidosis granulation tissue could synthesize and release 1,25-dihydroxycholecalciferol, leading to hypercalcinemia. Moreover, 1,25-dihydroxycholecalciferol levels were related to the expression of ICAM-1. In 1999, Braun et al. [30] applied 1,25-dihydroxycholecalciferol to stimulate the accumulation of alveolar macrophages in healthy individuals and patients with sarcoidosis and found that this compound enhanced the expression of ICAM-1, indicating that sarcoidosis may stimulate the synthesis of ICAM-1 through granuloma-secreted 1,25-dihydroxycholecalciferol. ICAM-1 is also upregulated in fibrosis in chronic obstructive pulmonary disease (COPD), indicating its possible function in the enhancement of the immune response and promotion of fibrosis [31]. Therefore, elevated serum ICAM-1 levels may be responsible for inflammation and the formation of granulomas, which we will investigate in our future studies.

In summary, we observed 2 new protein targets as potential regulators of granuloma formation due to their interactions with the immune system. The molecular mechanisms through which these proteins mediate sarcoidosis are not yet known. For the first time, our study evaluated the potential of evaluating leptin and sICAM-1 for clinical diagnosis and determined the cutoff values of these targets for the differential diagnosis of sarcoidosis and tuberculosis. The combination of BMI-leptin and ICAM-1 had remarkable sensitivity of 86.5% and specificity of 73.1%, suggesting the evaluation of this combination as a potential method for differential diagnosis. Further prospective controlled studies are required to verify these results, and we will continue to develop diagnosis kits with multiple differentially expressed proteins.

## Supporting Information

S1 File Detailed description of how we selected the 30 cytokines and chemokines evaluated in the study. (DOCX)

S1 Table Raw data and *P* value of 120 kinds of proteins and cytokines in the serum from SA (n = 3) and HC (n = 2) patients. (DOCX)
