# Supplementary material for: Screening for Differentially Expressed Proteins Relevant to the Differential Diagnosis of Sarcoidosis and Tuberculosis
Source: PLoS One. 2015 Sep 14;10(9):e0132466. doi: 10.1371/journal.pone.0132466 (PMC4569088; doi:10.1371/journal.pone.0132466)
Supplement: S1 File — (DOCX) [file pone.0132466.s001.docx]

Supporting information：

detailed description of how we selected 30 kinds of cytokines and chemokines

First, We orderd a protein chip which contains 120 kind of proteins and cytokines (Table S1)，and we test the serum of 3 sarcoidosis（n=3） and health control（n=2）. There were eleven proteins (MCP-1、IFN-gamma、IL-6、LIGHT、TGF-beta 1、IL-1alpha、GM-CSF、IL-7、Leptin、Eotaxin、PDGF-BB) were different between sarcoidosis and health controls (Table S1: *SA vs. HC *P*<0.05)

Then,considering the sample size is so small and the previous literature about sarcoidosis has reported the cytokine (see “discussion”section), we added aditional 19 factors ( Flt-3L、G-CSF、IL-10、IL-12 p40、IL-12 p70、IL-13、IL-15、IL-17、IL-1b、IL-4、MCP-2、

MIG、NT-3、ICAM-1、BMP-7、IL-22、IL-9、MMP-7、TSLP.) into our new protein chip which contains 30 kinds of protein and cytokines finally.
