# Supplementary material for: Screening for Differentially Expressed Proteins Relevant to the Differential Diagnosis of Sarcoidosis and Tuberculosis
Source: PLoS One. 2015 Sep 14;10(9):e0132466. doi: 10.1371/journal.pone.0132466 (PMC4569088; doi:10.1371/journal.pone.0132466)
Supplement: S1 Table — (DOCX) [file pone.0132466.s002.docx]

Supporting information：Table S1

Table S1． Raw data and *P* value of 120 kinds of proteins and cytokines in Serum of SA（n=3） and HC（n=2）

|  | SA-1 | SA-2 | SA-3 | HC-1 | HC-2 | *P* value（SA vs. HC） |
| --- | --- | --- | --- | --- | --- | --- |
| Angiogenin | 59,559 | 49,848 | 61,829 | 55,846 | 59,577 | 0.906287 |
| BDNF | 1,970 | 692 | 1,040 | 350 | 932 | 0.351007 |
| BLC | 282 | 245 | 246 | 250 | 914 | 0.282673 |
| BMP-4 | 224 | 231 | 190 | 155 | 1,986 | 0.298993 |
| BMP-6 | 352 | 321 | 227 | 209 | 342 | 0.750318 |
| CK beta 8-1 | 129 | 145 | 98 | 129 | 2,346 | 0.270613 |
| CNTF | 303 | 546 | 329 | 364 | 413 | 0.968788 |
| EGF | 6,226 | 1,970 | 8,203 | 301 | 2,738 | 0.218334 |
| Eotaxin | 231 | 170 | 229 | 330 | 316 | **0.023287*** |
| Eotaxin-2 | 627 | 290 | 358 | 327 | 1,689 | 0.347106 |
| Eotaxin-3 | 195 | 124 | 534 | 168 | 780 | 0.547486 |
| FGF-6 | 1,256 | 1,126 | 1,372 | 1,101 | 1,145 | 0.260662 |
| FGF-7 | 871 | 563 | 833 | 332 | 514 | 0.102116 |
| Flt-3 Ligand | 953 | 883 | 709 | 696 | 673 | 0.180289 |
| Fractalkine | 180 | 133 | 107 | 179 | 4,856 | 0.265992 |
| GCP-2 | 373 | 154 | 212 | 174 | 1,599 | 0.319709 |
| GDNF | 472 | 256 | 333 | 281 | 1,550 | 0.326109 |
| GM-CSF | 1,974 | 1,878 | 1,620 | 771 | 1,251 | **0.036375*** |
| I-309 | 218 | 342 | 158 | 127 | 1,621 | 0.340325 |
| IFN-gamma | 2,007 | 1,785 | 1,583 | 884 | 894 | **0.010682*** |
| IGFBP-1 | 1,390 | 497 | 749 | 466 | 952 | 0.690783 |
| IGFBP-2 | 5,540 | 5,521 | 4,895 | 6,712 | 4,905 | 0.548935 |
| IGFBP-4 | 333 | 227 | 171 | 326 | 1,395 | 0.223427 |
| IGF-I | 210 | 152 | 85 | 84 | 2,349 | 0.296003 |
| IL-10 | 2,813 | 1,900 | 2,290 | 1,029 | 1,357 | 0.051395 |
| IL-13 | 4,052 | 3,318 | 2,405 | 1,400 | 1,569 | 0.064164 |
| IL-15 | 3,195 | 3,313 | 1,363 | 1,272 | 1,738 | 0.272127 |
| IL-16 | 899 | 245 | 424 | 340 | 485 | 0.69759 |
| IL-1alpha | 3,483 | 2,552 | 2,700 | 1,348 | 1,427 | **0.026644*** |
| IL-1beta | 290 | 119 | 157 | 175 | 2,434 | 0.278159 |
| IL-1ra | 780 | 517 | 376 | 338 | 1,300 | 0.550884 |
| IL-2 | 1,622 | 1,239 | 1,333 | 806 | 979 | 0.05239 |
| IL-3 | 530 | 429 | 432 | 278 | 541 | 0.646 |
| IL-4 | 336 | 312 | 286 | 162 | 337 | 0.429227 |
| IL-5 | 1,841 | 889 | 1,349 | 529 | 797 | 0.155051 |
| IL-6 | 1,864 | 1,943 | 1,775 | 927 | 1,279 | **0.013705*** |
| IL-7 | 1,778 | 1,089 | 1,689 | 442 | 648 | **0.043692*** |
| Leptin | 394 | 1,403 | 741 | 3,143 | 3,311 | **0.008668*** |
| LIGHT | 485 | 477 | 536 | 267 | 355 | **0.019092*** |
| MCP-1 | 2,177 | 1,991 | 2,310 | 863 | 1,050 | **0.003216*** |
| MCP-2 | 1,312 | 231 | 325 | 340 | 1,489 | 0.669471 |
| MCP-3 | 2,036 | 1,307 | 1,547 | 554 | 1,184 | 0.127325 |
| MCP-4 | 313 | 225 | 223 | 156 | 3,055 | 0.299841 |
| M-CSF | 400 | 239 | 412 | 361 | 889 | 0.282134 |
| MDC | 3,898 | 3,510 | 1,332 | 2,027 | 2,165 | 0.486488 |
| MIG | 2,028 | 1,300 | 1,540 | 721 | 1,098 | 0.104768 |
| MIP-1-delta | 4,066 | 597 | 1,015 | 783 | 3,897 | 0.822283 |
| MIP-3-alpha | 99 | 94 | 100 | 89 | 1,987 | 0.275643 |
| NAP-2 | 5,621 | 3,594 | 5,121 | 3,564 | 5,379 | 0.78644 |
| NT-3 | 373 | 338 | 577 | 536 | 557 | 0.310108 |
| PARC | 743 | 1,450 | 1,679 | 901 | 1,025 | 0.436648 |
| PDGF-BB | 34,861 | 31,254 | 35,274 | 7,628 | 5,391 | **0.000673*** |
| RANTES | 49,146 | 36,658 | 41,950 | 29,167 | 38,891 | 0.243707 |
| SCF | 472 | 138 | 214 | 127 | 1,129 | 0.437704 |
| SDF-1 | 442 | 247 | 220 | 162 | 4,624 | 0.298323 |
| TARC | 4,888 | 9,533 | 1,416 | 414 | 978 | 0.229132 |
| TGF-beta 1 | 3,235 | 2,487 | 3,163 | 1,485 | 1,636 | **0.020672*** |
| TGF-beta 3 | 287 | 200 | 244 | 210 | 365 | 0.552905 |
| TNF-alpha | 1,912 | 1,363 | 1,403 | 719 | 1,041 | 0.077827 |
| TNF-beta | 2,055 | 1,571 | 1,510 | 871 | 1,186 | 0.072849 |
| Acrp30 | 21,861 | 32,913 | 30,372 | 38,327 | 36,226 | 0.1355601 |
| AgRP | 310 | 180 | 93 | 151 | 2,746 | 0.2867886 |
| Angiopoietin-2 | 642 | 545 | 208 | 216 | 1,168 | 0.6031645 |
| Amphiregulin | 450 | 525 | 111 | 108 | 1,919 | 0.4178275 |
| Axl | 936 | 1,482 | 815 | 1,177 | 4,933 | 0.2592647 |
| bFGF | 238 | 174 | 76 | 90 | 1,042 | 0.3441104 |
| b-NGF | 263 | 229 | 50 | 62 | 475 | 0.6524914 |
| BTC | 33 | 20 | 0 | 39 | 2,394 | 0.2654899 |
| CCL-28 | 90 | 153 | 50 | 51 | 850 | 0.3255914 |
| CTACK | 766 | 951 | 495 | 612 | 1,564 | 0.4388064 |
| Dtk | 45 | 55 | 17 | 52 | 4,202 | 0.2698327 |
| EGF-R | 1,423 | 1,350 | 1,278 | 1,470 | 1,240 | 0.9646045 |
| ENA-78 | 483 | 433 | 180 | 209 | 1,056 | 0.4873838 |
| Fas/TNFRSF6 | 850 | 801 | 543 | 550 | 1,194 | 0.6360418 |
| FGF-4 | 256 | 244 | 70 | 59 | 1,217 | 0.3824698 |
| FGF-9 | 396 | 428 | 179 | 45 | 863 | 0.7330475 |
| GCSF | 518 | 409 | 266 | 176 | 340 | 0.3029958 |
| GITR-Ligand | 154 | 152 | 55 | 11 | 1,237 | 0.3528379 |
| GITR | 600 | 488 | 145 | 86 | 1,826 | 0.4766598 |
| GRO | 1,767 | 1,495 | 1,604 | 778 | 1,256 | 0.0600875 |
| GRO-alpha | 861 | 1,173 | 862 | 521 | 1,103 | 0.590513 |
| HCC-4 | 835 | 278 | 187 | 208 | 1,345 | 0.5401568 |
| HGF | 363 | 171 | 94 | 48 | 2,614 | 0.3277995 |
| ICAM-1 | 169 | 174 | 325 | 86 | 10,061 | 0.2830775 |
| ICAM-3 | 19 | 23 | 2 | 0 | 1,655 | 0.2791712 |
| IGFBP-3 | 234 | 156 | 54 | 74 | 3,137 | 0.2922973 |
| IGFBP-6 | 1,410 | 1,432 | 1,050 | 1,280 | 4,769 | 0.2789914 |
| IGF-I SR | 162 | 128 | 49 | 75 | 4,525 | 0.2791066 |
| IL-1 R4/ST2 | 528 | 487 | 126 | 187 | 556 | 0.9701994 |
| IL-1 RI | 185 | 234 | 41 | 13 | 2,892 | 0.3135781 |
| IL-11 | 159 | 307 | 48 | 35 | 25 | 0.2408365 |
| IL-12 p40 | 334 | 338 | 138 | 112 | 3,131 | 0.3170239 |
| IL-12 p70 | 586 | 581 | 213 | 88 | 2,435 | 0.4336984 |
| IL-17 | 177 | 210 | 49 | 50 | 75 | 0.2861739 |
| IL-2 Rapha | 532 | 668 | 43 | 61 | 498 | 0.6776503 |
| IL-6 R | 7,159 | 5,584 | 4,732 | 5,041 | 5,436 | 0.5729892 |
| IL-8 | 375 | 342 | 358 | 232 | 390 | 0.4825865 |
| I-TAC | 211 | 201 | 80 | 60 | 240 | 0.8802449 |
| Lymphotactin | 100 | 27 | 0 | 2 | 2,305 | 0.2864203 |
| MIF | 189 | 169 | 162 | 43 | 3,436 | 0.3034671 |
| MIP-1alpha | 418 | 232 | 9 | 15 | 1,985 | 0.3747034 |
| MIP-1beta | 2,272 | 2,577 | 1,971 | 427 | 3,339 | 0.7478531 |
| MIP-3beta | 908 | 1,352 | 7 | 12 | 26 | 0.2446486 |
| MSP-alpha | 1,448 | 664 | 456 | 433 | 1,121 | 0.876965 |
| NT-4 | 70 | 66 | 0 | 5 | 3,531 | 0.2813118 |
| Osteoprotegerin | 166 | 113 | 206 | 96 | 3,414 | 0.2881602 |
| Oncostatin M | 321 | 342 | 175 | 65 | 1,037 | 0.5146807 |
| PIGF | 410 | 247 | 90 | 85 | 1,846 | 0.3616283 |
| sgp130 | 1,988 | 1,876 | 1,408 | 1,411 | 2,836 | 0.5713185 |
| sTNF RII | 1,029 | 2,147 | 313 | 637 | 2,002 | 0.8665327 |
| sTNF-RI | 372 | 117 | 46 | 48 | 1,875 | 0.3405238 |
| TECK | 93 | 65 | 23 | 51 | 2,660 | 0.2752322 |
| TIMP-1 | 1,595 | 1,914 | 2,851 | 1,257 | 3,051 | 0.9700204 |
| TIMP-2 | 1,164 | 1,160 | 419 | 2,316 | 5,070 | 0.0814174 |
| Thrombopoietin | 409 | 245 | 46 | 85 | 883 | 0.4990379 |
| TRAIL R3 | 3,237 | 3,216 | 598 | 3,570 | 4,432 | 0.2551514 |
| TRAIL R4 | 440 | 216 | 104 | 72 | 1,579 | 0.3936905 |
| uPAR | 726 | 523 | 309 | 267 | 660 | 0.810141 |
| VEGF | 250 | 263 | 73 | 21 | 2,138 | 0.3462091 |
| VEGF-D | 272 | 234 | 64 | 35 | 3,440 | 0.3107325 |

* SA vs. HC *p*<0.05.
